# Trans-thoracic Extension of a Lung Abscess With a Pleurocutaneous Fistula: A Report of a Rare Case

**DOI:** 10.7759/cureus.103344

**Published:** 2026-02-10

**Authors:** Ricardo Velho, Henrique Borges, Odete Duarte, Christine C Paiva, Tiago Jorge Costa, Manuel Maia, Bernardo Belchior, João Coelho, José Brito, Mariana Lourenço, Laura Cunha Dias, Ana Sofia Teixeira

**Affiliations:** 1 Internal Medicine, Unidade Local de Saúde de Coimbra, Coimbra, PRT; 2 Nephrology, Unidade Local de Saúde do Algarve, Faro, PRT

**Keywords:** chest wall invasion, computed tomography, lung abscess, pleurocutaneous fistula, trans-thoracic extension

## Abstract

Lung abscesses are localized collections of pus within the pulmonary parenchyma, usually arising from necrotizing infection and commonly occurring in patients with risk factors such as immunosuppression or aspiration. Typical complications include pleural effusion, empyema and bronchopleural fistula. Direct invasion of the chest wall by a lung abscess is an exceptionally rare complication, with most reported cases related to tuberculous empyema (empyema necessitans) rather than being related to the primary pulmonary infection. The authors report a case of a 42-year-old male patient with chronic hepatitis C, former intravenous drug use, and active smoking, who presented with right-sided pleuritic chest pain, productive cough, dyspnea and progressive swelling of the right anterior chest wall. Computed tomography revealed a large multiloculated abscess in the right upper lobe, measuring 13 x 8 x 7.5 cm, with direct trans-thoracic extension through the intercostal space and invasion of the right pectoralis major muscle, consistent with a pleurocutaneous fistula. Microbiological studies identified methicillin-sensitive *Staphylococcus aureus* as the causative organism. The patient was successfully treated with surgical drainage and prolonged antibiotic therapy, resulting in complete clinical and radiological resolution. This report presents an extremely rare presentation of lung abscess with pleurocutaneous fistulization in the absence of empyema or tuberculosis, emphasizing the critical role of computed tomography in diagnosis and management.

## Introduction

A lung abscess is a cavity within the lung parenchyma containing pus or necrotic tissue. It is often a result of an infection that was left untreated and progressed to pneumonitis and tissue necrosis. It often arises in immunocompromised patients and patients at high risk for aspiration. Other causes of lung abscesses include bronchial obstruction by a tumor, foreign body, enlarged lymph node, or congenital malformation [1]. Symptoms of lung abscesses include fever, productive cough, chest pain, and night sweats. The diagnosis of lung abscesses includes computed tomography scans and thoracic ultrasound imaging. Microbiological studies, such as sputum examination and bronchoscopy, are essential to study the underlying cause of lung abscesses. Treatment of lung abscesses is based on antibiotic therapy and surgical or percutaneous interventions [2,3]. A rare complication of lung abscess is direct invasion of the chest wall. This phenomenon is classically described as empyema necessitans when it arises from chronic pleural empyema, most often in the setting of tuberculosis, with extension of the purulent material through the thoracic wall into the subcutaneous tissues. In contrast, isolated trans-thoracic extension of a primary pulmonary abscess, without associated empyema or tuberculosis, is exceedingly rare and has been reported only sporadically in the literature [4,5]. We report a case of a lung abscess with direct fistulization through the intercostal space into the right pectoralis major muscle, highlighting its clinical presentation, imaging findings, and management.

## Case presentation

Our report describes a 42-year-old man who presented to the emergency department after seven days of progressive symptoms, including right-sided pleuritic chest pain, productive cough with yellow sputum, dyspnea, and progressive swelling of the right anterior chest wall. No fever was reported. His past medical history included chronic, untreated hepatitis C, former intravenous drug abuse, and 20 pack-year smoking history. He was chronically medicated with buprenorphine. He lived in his own home with good hygienic conditions, had contact with one domestic dog, no recent travel, and no known history of tuberculosis or contact with individuals with tuberculosis. There was no documented recent weight loss. On physical examination, he was pale, thin, and had a painful deformity of the anterior chest wall, without signs of erythema or local warmth. He was afebrile (37.2 ºC) and had a peripheral oxygen saturation of 99% on room air. Other vital signs were within normal limits. He had decreased breath sounds in the right upper lung field. Laboratory results showed increased white blood cell count (with neutrophilia), mild elevation of liver enzymes on admission (in the context of known chronic hepatitis C), and elevated C-reactive protein (Table 1).

**Table 1:** Laboratory results on admission. L: liter; dL: deciliter; U: units; mg: miligrams

Parameter	Results on admission	Normal range
White blood cell count	11.7 x 10^9^/L	3.9 – 10.2 x 10^9^/L
Neutrophils	9.69 x 10^9^/L	1.5 – 7,7 x 10^9^/L
Hemoglobin	13.4 g/dL	13.5 – 17.5 g/dL
Platelets	190 x 10^9^/L	150 – 450 x 10^9^/L
Lactate dehydrogenase	311 U	< 248 U/L
Alanine aminotransferase	46	< 45 U/L
Aspartate aminotransferase	73	< 35 U/L
C-reactive protein	11.3 mg/dL	< 0.5 mg/dL

**Figure 1:**
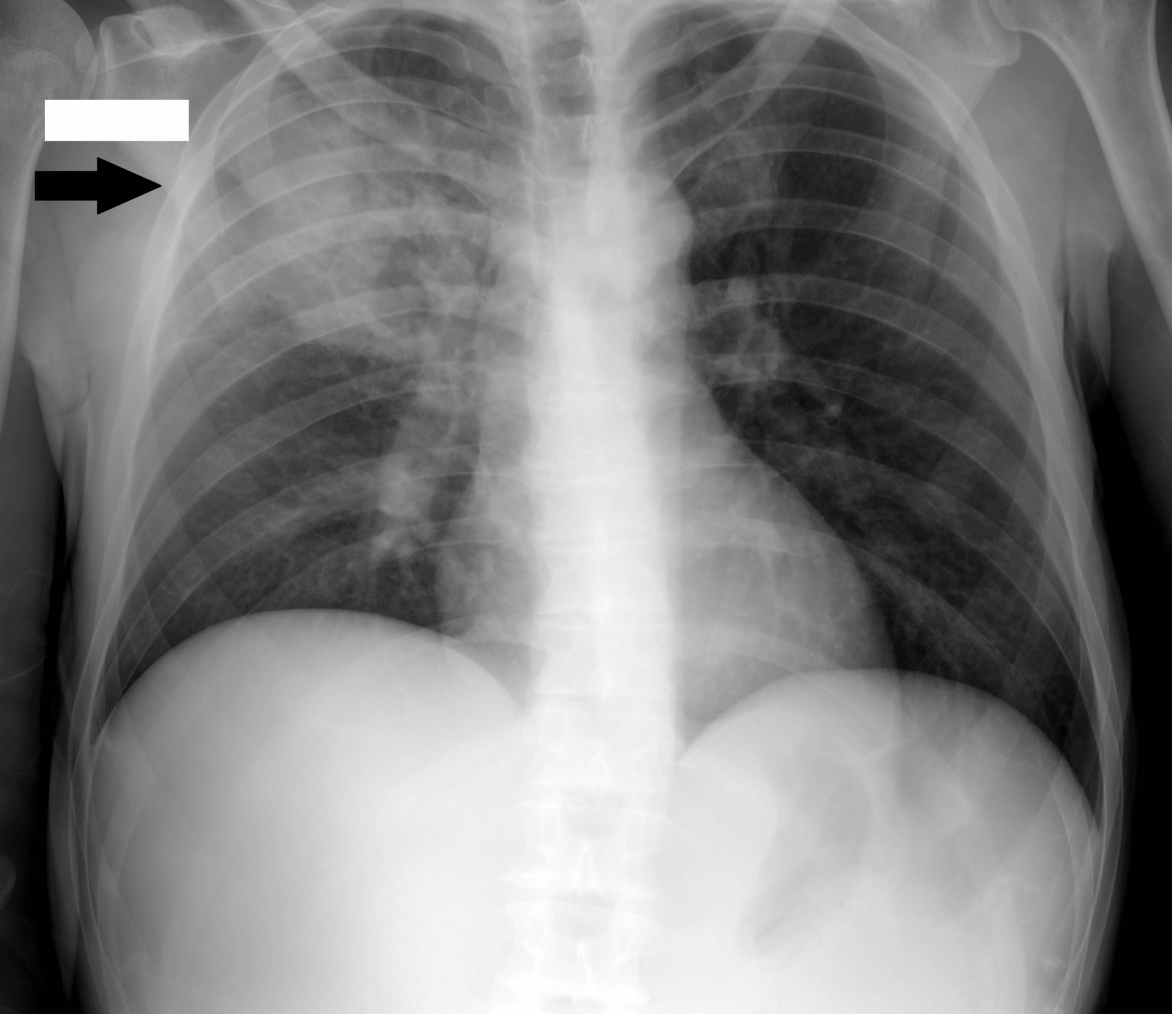
Chest radiography showing a large opacity in the right upper pulmonary field.

**Figure 2:**
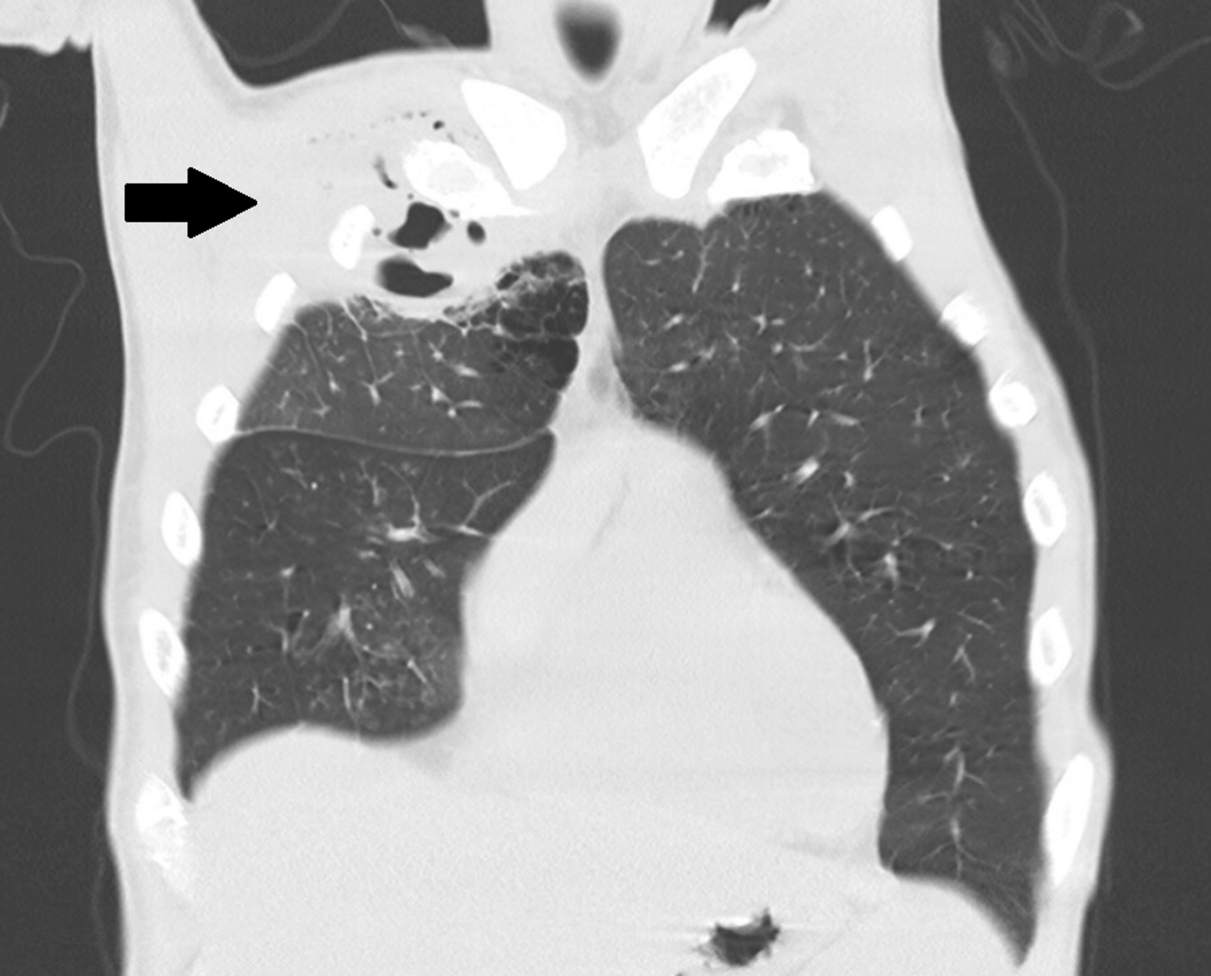
Coronal contrast-enhanced chest CT (lung window) demonstrating a large multiloculated gas-containing abscess in the right upper lobe, with loss of the normal plane between the lung and the anterior chest wall, consistent with trans-thoracic extension.

**Figure 3:**
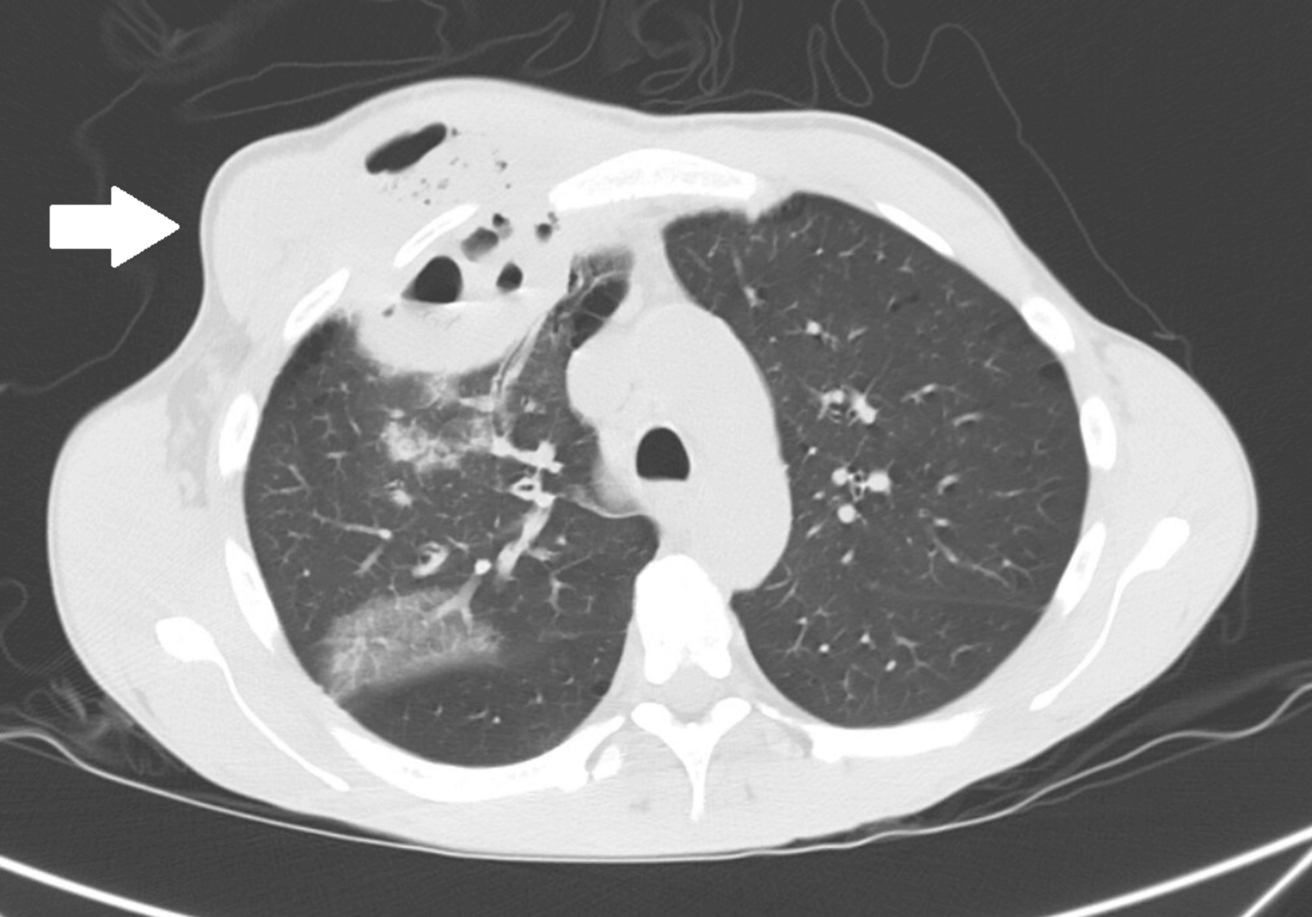
Axial chest CT (lung window) showing a cavitary lesion with internal gas in the right upper lobe, associated with surrounding inflammatory changes, confirming the pulmonary origin of the abscess.

**Figure 4:**
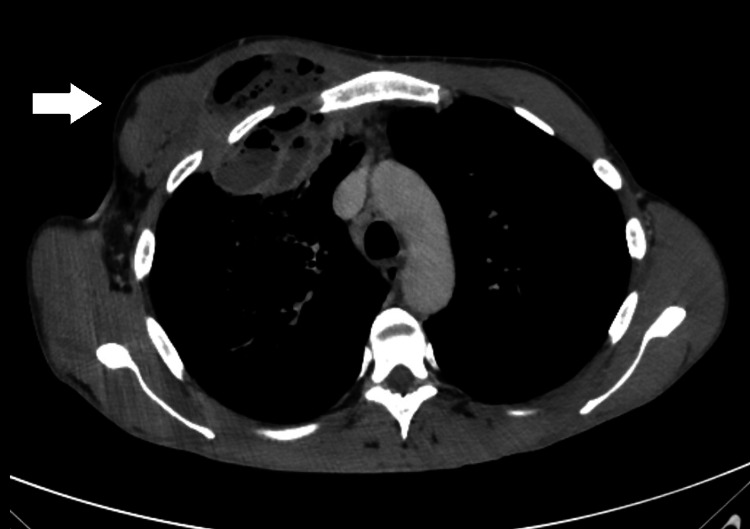
Axial contrast-enhanced chest CT (soft tissue window) demonstrating extension of the infectious process through the intercostal space with formation of an extra-thoracic collection involving the right anterior chest wall, compatible with a pleurocutaneous fistula.

Chest radiography showed a large opacity without defined borders in the right upper pulmonary field (Figure 1). A computed tomography scan reported a large heterogeneous, multiloculated collection in the right anterior hemithorax, with peripheral enhancement and containing gas, measuring approximately 13 x 8 x 7.5 cm. This lesion extended from the pulmonary parenchyma of the right upper lobe to the soft tissues outside the thoracic cavity, through fistulization across the intercostal space. This lesion was collected predominantly posterior to the right pectoralis major muscle, although already invading it (Figures 2-4).

He was tested for human immunodeficiency virus (HIV), which was negative. Blood cultures, sputum bacterial culture, and tuberculosis workup were obtained, including sputum examination for acid-fast bacilli, mycobacterial culture, and nucleic-acid amplification testing for *Mycobacterium tuberculosis*. The results are depicted in Table 2.

**Table 2:** Microbiological studies and results.

Microbiological study	Results
Blood cultures (2)	Negative
Sputum bacterial culture	Methicillin-sensitive *Staphylococcus aureus*
Sputum examination for acid-fast bacilli	Negative
Mycobacterial culture	Negative
Nucleic acid amplification testing for *Mycobacterium tuberculosis*	Negative

Before the results of the cultures were available, empirical antibiotic therapy was initiated with piperacillin/tazobactam, vancomycin, and metronidazole to provide broad coverage for severe polymicrobial infection, including Gram-positive organisms, Gram-negative bacilli, anaerobes, and methicillin-resistant *Staphylococcus aureus*, given the presence of a large multiloculated gas-containing collection with extension into the chest wall.

Thoracic surgery performed open drainage of the chest wall abscess shortly after admission, yielding approximately 120 milliliters of thick purulent fluid. Culture of the abscess fluid grew methicillin-sensitive *Staphylococcus aureus*, prompting antibiotic de-escalation to intravenous cefazolin for 19 days. The patient improved steadily and was discharged on day 23 with an additional oral course of cefuroxime, for a total of 50 days. The isolation of methicillin-sensitive *Staphylococcus aureus* from both sputum and abscess fluid supported a pulmonary origin of the infection. At three-month follow-up, repeat chest computed tomography confirmed complete resolution of the pulmonary abscess and chest wall involvement.

## Discussion

Pleurocutaneous fistulas are exceedingly rare and are often a result of chronic empyema, classically associated with tuberculosis. This condition is called empyema necessitans, and it is a condition nowadays considered unusual, with most contemporary cases occurring in immunocompromised or tuberculous patients [6]. Reports of lung abscesses, rather than empyemas, extending through the thoracic wall are even less common and exist primarily as isolated case descriptions [7]. The mechanism in the present case is suggested by the results of computed tomography imaging. The patient most likely developed a necrotizing Methicillin-sensitive *Staphylococcus aureus* lung abscess, which ruptured through the visceral pleura and dissected through the intercostal space, invading the pectoralis major muscle. The absence of pneumothorax or pleural air reinforces that this was not a bronchopleural fistula, which requires communication between the bronchial tree and the pleural space. Computed tomography imaging is of utmost importance in these cases to delineate fistula tracts, identify multiloculated abscesses with gas, differentiate infection from chest wall tumors, and help plan drainage or surgery. Management of these cases typically combines drainage of the extra-thoracic collection and antibiotic therapy targeted to the causative organism [2,3]. Methicillin-sensitive *Staphylococcus aureus* infections generally respond well to first-generation cephalosporins, as in this case. Cases similar to this are scarcely reported in scientific literature, which may be explained by the rarity of this event. Most of the cases of invasion of the chest wall are caused by empyema necessitans, which is not the case in this presentation, as there was no empyema and no tuberculosis infection [8-11]. Unlike empyema necessitans, this case represents a direct trans-thoracic extension of a primary pulmonary abscess, without pleural empyema or tuberculosis. This fact emphasizes the rarity of this case.

## Conclusions

This case report illustrates an exceptionally rare case of a pleurocutaneous fistula due to trans-thoracic extension of a lung abscess, in the absence of empyema or tuberculosis, a scenario reported only sporadically in the modern literature. In this case, computed tomography imaging was crucial to the diagnosis. Prompt recognition of this unusual presentation allowed timely combined surgical and antimicrobial treatment, resulting in complete clinical and radiological resolution. Clinicians should be aware of this rare pattern of extra-thoracic extension when evaluating chest wall masses in patients with respiratory symptoms and risk factors for pulmonary infection.
